# Heat-Induced Interaction of Milk Proteins: Impact on Yoghurt Structure

**DOI:** 10.1155/2021/5569917

**Published:** 2021-09-22

**Authors:** Md Asaduzzaman, Md Sultan Mahomud, Mohammod Enamul Haque

**Affiliations:** ^1^Faculty of Science and Technology, Free University of Bolzano, Piazza Università 1, 39100 Bolzano, Italy; ^2^Department of Food Engineering and Technology, Hajee Mohammad Danesh Science and Technology University, Dinajpur 5200, Bangladesh; ^3^Department of Animal Nutrition, Bangladesh Agricultural University, Mymensingh 2202, Bangladesh; ^4^Bangladesh Milk Producers' Cooperative Union Ltd., Dhaka 1216, Bangladesh

## Abstract

Heating milk for yoghurt preparation has a significant effect on the structural properties of yoghurt. Milk heated at elevated temperature causes denaturation of whey protein, aggregation, and some case gelation. It is important to understand the mechanism involved in each state of stabilization for tailoring the final product. We review the formation of these complexes and their consequence on the physical, rheological, and microstructural properties of acid milk gels. To investigate the interactions between denatured whey protein and casein, the formation of covalent and noncovalent bonds, localization of the complexes, and their impact on ultimate gelation and final yoghurt texture are reviewed. The information regarding this fundamental mechanism will be beneficial to develop uniform quality yoghurt texture and potential interest of future research.

## 1. Introduction

Yoghurt is one of the popular fermented milk products in the world, widely perceived as a healthy food by consumers as it contains basic nutrients, bioactive compounds, and health-promoting probiotics. For instance, plant extracts, gut-friendly prebiotics, omega-3 fatty acid, vitamin, minerals, and fibers are often incorporated as functional ingredients in yoghurt [1]. During the fermentation process, starter cultures reduce the milk pH to the isoelectric point of casein (pH 4.6); as a result, casein micelle becomes unstable and coagulates and formed three-dimensional protein networks in which whey is entrapped [2, 3]. Milk heated at an elevated temperature (>70°C) causes denaturation of whey protein and favors the formation of the disulfide bond between *κ*-casein and denatured whey proteins and ultimate gel structure [4, 5]. Several factors influence this bond formation such as the temperature, heating time, protein concentration, and pH of heated milk [6, 7]. For instance, during heating milk, the tertiary structure of globular whey proteins is unfolded, and free thiol groups are exposed. Consequently, thiol (-SH)/disulphide (S-S) interchange reactions are promoted during the acidification of heated milk. Such covalent bonds contribute to the ultimate gel strength of yoghurt [5, 6, 8].

Several studies have described extensively the heat-induced denaturation and interaction of whey proteins in the skim milk with or without added whey protein. Depending on the milk pH, heat-induced denatured whey protein may partly be associated as casein micelle-bound complexes or as soluble whey protein (WP)/*κ*-casein (*κ*-Cn) complex [5, 9, 10]. These micelle-bound and/or soluble WP/*κ*-Cn complexes are the crucial factor to define the properties of yoghurt.

In this perspective, it is imperative to focus on the type of milk protein interactions and their effect on the functional properties of yoghurt gel. The objective of this review is to summarize the fundamental knowledge of the effect of casein micelle-bound and soluble WP/*κ*-Cn complex or whey protein-whey protein aggregation on the properties of yoghurt. The present review will discuss first the formation of whey protein-casein complexes, their localization in heated milk, and the impact on gel formation. Finally, the role of these protein complexes on the properties of yoghurt is reviewed which will provide some hands-on information for the yoghurt manufacturer.

## 2. Whey Protein-Casein Complex Formation in Heated Milk

**Figure 1:**
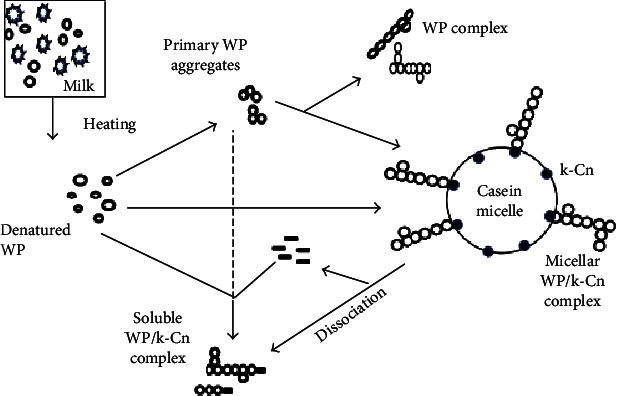
Schematic diagram of proposed pathway of the interaction between denatured whey protein and *κ*-casein in heated milk. WP: whey protein, *κ*-Cn: *κ*-casein (adapted from [36]).

Generally, milk used in yoghurt preparation is subjected to prolong heat treatment not only to destroy microbial growth but also to obtain improved body texture of the gel. Heat treatment is the most commonly used processing tools to enhance the denaturation of whey proteins and their interaction with casein micelles [4, 5, 11–13]. One of the interactions of interest is *β*-lactoglobulin-casein micelle complex formation. In practice, a different temperature-time combination such as 85°C for 30 min, 90-95°C for 5-10 min, or 115°C for 3 s is applied in milk used for yoghurt production which causes denaturation of whey proteins and promote the complex formation between casein and whey protein via covalent bonds and/or hydrophobic interactions (Figure 1) [5, 13, 14].

In heated milk, denatured whey protein interacted with *κ*-casein of the casein micelles or free *κ*-casein in the serum phase which acts as bridging material to interact with other denatured whey proteins resulting in the formation of complex protein networks [4, 15]. Upon heating, covalent bonds are formed between *β*-lactoglobulin (*β*-Lg) and *κ*-casein through -SH/S-S interchange reactions [14, 16], and there is some evidence that *α*-lactalbumin (*α*-La) is also associated with these complexes [17]. Besides, bovine serum albumin (BSA), lactoferrin, and *α*_s2_-caseins, having free thiol group, may also participate in this complex formation via -SH/S-S interchange reactions [14, 18], whereas immunoglobulins are partially involved in these complexes through hydrophobic interactions [19]. Therefore, it is evident that the formation of WP/*κ*-Cn complexes is mostly the result of either through -SH/S-S interchange reactions or hydrophobic interactions. A comprehensive understanding of such complex formations might be helpful to study the interaction of casein from alternative sources.

### 2.1. Covalent Bond Formation

The major whey protein, *β*-lactoglobulin, represents about 50% of the whey proteins and 12% of total milk proteins, which exists as a dimer at room temperature but converted into monomers at a higher temperature. It contains two disulfide bonds between Cys106-Cys119 and Cys66-Cys160, and one free thiol group (Cys121) is of great importance as it is involved in the reaction with other proteins. Other whey proteins, for example, *α*-lactalbumin, have four S-S bonds between Cys6-Cys120, Cys28-Cys111, Cys61-Cys77, and Cys73-Cys91 but does not have any free thiol group. The tertiary structure of BSA reveals three equal-sized globular domains. It contains one free thiol group (Cys34) same as *β*-lactoglobulin as well as seventeen S-S bonds [20]. Of the casein proteins, both *κ*-casein and *α*_s2_-casein have two thiol groups; therefore, both can participate in -SH/S-S interchange reactions. Early studies proposed that there may be an interaction between whey protein and casein [21]. However, the first conclusive evidence of *β*-Lg/casein micelle complex formation has been presented early in “the ‘70s” [22]. Sawyer and his coworker suggested that the free thiol group of *β*-lactoglobulin is involved in the interaction and form disulfide bridges between *κ*-casein and *β*-lactoglobulin [14, 23].

The exposed thiol groups of *β*-lactoglobulin are responsible for the covalent bond formation. However, aggregation of whey proteins is not only the result of reactive free thiol groups but also disulfide within the structure of the proteins. The participation of the different thiol groups depends on their location in the native structure of the protein. Thiol groups located on the surface are more prone to disulfide bond formation than internally located ones [18, 24]. At elevated temperature, one disulfide bond (Cys66-Cys160) and free cysteine (thiol group, -SH) of the *β*-lactoglobulin monomer are exposed which initiate -SH/S-S interchange reaction [14, 25, 26]. The covalent intermolecular disulfide bond is formed between Cys160 of *β*-lactoglobulin with *κ*-casein on the surface of casein micelles [27] or in the serum phase [24] and with the cysteine of other *β*-lactoglobulin molecules rather than intermolecular bound with Cys66 [28–30].

Monomeric *β*-lactoglobulin is responsible for the formation of intramolecular and intermolecular bonds. Cys121 of *β*-lactoglobulin plays an important role in initiating the -SH/S-S interchange reaction. [30] reported that the disulfide bond between monomers is more extensive and even trimer also formed by connecting the tree adjacent cysteines (Cys106/Cys119/Cys121). However, other also reported that at initial stage, Cys121 is involved in intramolecular disulfide bond formation with other two cysteine residues of *β*-lactoglobulin monomer; therefore, Cys160 and Cys66 are implicated for the intermolecular disulfide bond formation [24, 27, 30], whereas Cys106 of *β*-lactoglobulin is less accessible for the formation of the intermolecular disulfide bond due to remain buried inside the inner parts of the proteins [24, 29].

The disulfide between Cys6-Cys120 and Cys28-Cys111 of *α*-lactalbumin is located on the surface of the protein. Upon heating the bond is cleaved, and free thiol groups are readily accessible to -SH/S-S covalent bond formation except for Cys28; it becomes inaccessible for aggregation reactions due to inward retraction [20, 31]. In *α*-lactalbumin, Cys6, Cys120, Cys61, and Cys111 are mostly involved in disulfide bond formation with *β*-lactoglobulin, whereas Cys91, Cys77, Cys73, and Cys28 do not involve due to their inaccessible location in native protein [30]. Cys121 of *β*-lactoglobulin is known to initiate -SH/S-S interchange reaction with Cys6, Cys61, and Cys 120 of *α*-lactalbumin, whereas Cys66 and Cys160 of *β*-lactoglobulin are mostly involved in forming a disulfide bond with Cys111 of *α*-lactalbumin. However, intramolecular disulfide bond of *α*-lactalbumin is more favorable than the intermolecular disulfide bond between *α*-lactalbumin and *β*-lactoglobulin. An interaction between *α*-lactalbumin and *κ*-casein is also possible if there is still enough free reactive thiol present [14, 32, 33].

Upon heating, BSA also undergoes conformationally different monomer and expose free thiol group on the surface. This free thiol may react with BSA via -SH/S-S interchange reaction and form dimer, trimer, and so on. Free thiol of BSA may also involve in -SH/S-S interchange reaction with Cys6 or Cys120 of *α*-lactalbumin [34, 35]. In a mix solution, BSA is more effective than *β*-lactoglobulin to react with *α*-lactalbumin because a more covalent disulfide bond is formed between BSA/*α*-La than *β*-Lg/*α*-La combination [19, 34].

It is generally accepted that -SH/S-S interchange reactions occurred between the free thiol groups of *β*-lactoglobulin and the disulfide bonds of the *κ*-caseins (Figure 1), which remains in the boundary between the para-*κ*-casein (near micelle core) and the glycomacropeptide region (hairy brush). *β*-Lactoglobulin penetrates through the hairy layer of glycomacropeptide region [14, 32]. Early studies reported that *κ*-casein forms complex with intermediate species of self-aggregated *β*-lactoglobulin; therefore, intramolecular interaction of *β*-lactoglobulin monomer is a limiting factor when *κ*-casein present in the system [23, 36], while others also reported that the self-aggregation of *β*-lactoglobulin is not crucial to forming a complex with *κ*-casein [37]. Upon heating, the free thiol group of *β*-lactoglobulin initiates a series of -SH/S-S bonds with *κ*-casein in many possible pathways. The product formed depends on the ratio of both proteins [17, 24, 38]. Cys11 and Cys88 of *κ*-casein are randomly involved in -SH/S-S interchange reaction with Cys160 of *β*-lactoglobulin [24, 28].

In addition to *κ*-casein, *α*_s2_-casein may also undergo -SH/S-S interexchange reactions due to the presence of one disulfide bond in *α*_s2_-casein. The reaction is somehow less favorable because *α*_s2_-casein is present inside the casein micelle structure. However, when the casein micelles are disrupted, the disulfide bond of *α*_s2_-casein becomes free to participate -SH/S-S interexchange reactions with the free thiol group of other whey proteins [39, 40].

### 2.2. Electrostatic and Hydrophobic Interaction

Several studies demonstrated that the formation of intermolecular heat-induced noncovalent bonds (i.e., hydrophobic, electrostatic, and ionic interactions) is also formed between denatured whey protein and *κ*-casein [4, 20, 41, 42]. When covalent bonds cannot be built during heating, noncovalent bonds developed into heat-induced protein aggregations [34, 41]. The extent of noncovalent interactions and their contribution to the overall protein aggregations and gelation process become of increasing interest in the model systems. From the experiment of adding thiol blocking agent (N-ehthylmaleimide) in milk, it has been demonstrated that noncovalent bonds could occur followed by hydrophobic interaction and increase the total number of connections between protein complexes [14, 34, 43].

Upon heating, denatured *β*-lactoglobulin interacts with other proteins through intermolecular -SH/S-S interchange reactions and hydrophobic interactions. In the early stage of heating, *β*-lactoglobulin can interact with *κ*-casein via both hydrophobic and -SH/S-S interactions; nevertheless, this interaction depends on the type of proteins, temperature, and heating time of the system [38]. Others also reported that at the early stage, *β*-lactoglobulin interacts with *κ*-casein through hydrophobic attractions; after that, via -SH/S-S interchange reactions. Additionally, due to the absence of a free thiol group, *α*-lactalbumin may also form the *β*-Lg/*α*-La complex through noncovalent interaction [14, 44]. Some studies demonstrated that the role of ionic interactions on *β*-Lg/*α*-La/*κ*-Cn complex formation is lesser and questionable [45–47]. There is no clear information on whether hydrophobic or electrostatic interactions are dominant to form the *β*-Lg/*κ*-Cn complexes in heated milk systems.

### 2.3. Whey Protein Interaction with Micellar *κ*-Casein

Heat-induced denaturation of milk proteins reveals the thiol groups which are normally remained buried inside the native protein structure. These free thiol groups enhanced the interaction of the denatured whey protein to the surface of the casein micelles. The denatured whey protein reacts more with *κ*-casein in casein micelles rather than dissociated *κ*-casein in the serum [4, 48]. Although the hairy layer of *κ*-casein stabilizes casein micelles by electrostatic and steric repulsion, the gap between two *κ*-casein molecules is enough for whey protein entrance. Hence, whey protein can get access easily to individual *κ*-casein compared to *κ*-casein aggregates present in the serum phase [20, 28]. There are two limiting reaction mechanism involved in the association: first, exposer of the thiol groups of *β*-lactoglobulin and the second is the interaction of the thiol groups with the disulfide bond of *κ*-casein remaining on the surface of the casein micelles [36, 49]. Due to the absence of free thiol groups, *α*-lactalbumin may react with *κ*-casein only after -SH/S-S interchange reactions with *β*-lactoglobulin. When milk is heated at pH < natural milk pH, most of the denatured whey proteins tend to interact with *κ*-casein at the surface of the micelles and form micelle-bound complexes [5, 14, 50, 51]. The interaction between the whey proteins and *κ*-casein in casein micelles is better under slow heating than fast heating. At slow heating, small aggregated species of *β*-lactoglobulin are formed which easily penetrate the hairy layer of *κ*-casein and enhance better interactions. Casein micelle size also increases due to this complex [52–54].

### 2.4. Whey Protein Interaction with Dissociated *κ*-Casein

In the serum phase, denatured whey proteins also interact with *κ*-casein through -SH/S-S interchange reactions. The dissociation of *κ*-casein is of key importance to control the WP/*κ*-Cn complex formation. At an early stage of heating, *κ*-caseins dissociate from the casein micelles to the serum phase. Thereafter, free denatured whey proteins interact with soluble *κ*-casein via -SH/S-S interchange; subsequently, it forms the soluble denatured WP/*κ*-Cn complexes in the heated milk [6, 55, 56]. It has been reported that the dissociation of the *κ*-casein from the casein micelles is related to the pH of the heated milk [9]. Heating milk at pH > natural milk pH forms mostly soluble protein complex, and heating milk at pH< natural milk pH favors mostly micelle-bound complexes, whereas at pH ~ natural milk pH results in a balanced contribution from both complexes [5].

Addition of soluble *κ*-casein to the skim milk or when soluble *κ*-casein is heated excluding casein results in the formation of soluble WP/*κ*-Cn complexes more in serum phase than in micellar phase [14, 57]. It is also observed that the dissociation of the *κ*-casein from the casein micelles happens at lower temperatures than the denaturation temperature of whey proteins and also reached its peak before the denaturation of the whey proteins [4, 14]. During the cooling stage, the soluble *κ*-caseins which are not associated with the denatured whey proteins in the serum phase subsequently reassociated with the casein micelles [4].

## 3. Effect of Whey Protein/*κ*-Casein Complexes on Acid Gelation

The heat treatment of milk significantly improves the acid gelation properties of milk; hence, it is the most common processing step applied in yoghurt preparation. Adding starter culture in milk causes lactic acid production and subsequent decrease of pH from 6.8 to 4.6 which is the isoelectric point of casein micelles [58]. At this isoelectric point, the net negative charge of casein micelles reduced, electrostatic repulsions and steric stabilization decrease, then subsequent coagulation of casein results in the formation of three-dimensional gel networks [59–61]. Milk protein complexes with their new functionalities influence gelation point and gel network formation in yoghurt. Average pH value at which gelation start is ~4.8, whereas in presence of WP/*κ*-Cn complex, this value rises to ~5.4 [4, 52, 55]. The modified surface hydrophobicity of the WP/*κ*-Cn complex promotes the acid destabilization threshold due to the increase of net colloidal attraction. Isoelectric pH of WP/*κ*-Cn complex is also important for the onset of gelation points [4, 36]. Therefore, there is a scope for modulating the isoelectric pH and surface hydrophobicity of WP/*κ*-Cn complex to achieve the desired functionality. There are several methods which have been applied to modulate the functionality, such as protein biodiversity (i.e., the genetic variants of *β*-lactoglobulin and *κ*-caseins have different isoelectric pH and surface hydrophobicity) adding cysteine-containing globular protein, enzymatic modification, nonspecific fixation of charge ligands using anionic surfactants, and Millard reaction (attaching sugars with *ε*-amines of the WP/*κ*-Cn complex). A detail has been reviewed by Morand and his coworkers [62].

Although the addition of exogenous protein or denatured whey protein in milk increases the gelation point, natural *κ*-caseins of milk have great importance on the gelation mechanism. The early interaction between *κ*-caseins of casein micelles and heat-induced denatured whey protein influence acid gelation point. It has been reported that coheating of these two fractions result in an early gelation point [10, 36, 56]. The WP/*κ*-Cn complex that is present in the serum phase also binds on the surface of casein micelles and gives a compact structure of yogurt gel [6, 56]. The formation of the soluble WP/*κ*-Cn with a higher portion of *κ*-caseins is favored in higher pH than natural milk pH (~6.7). Acid gelation of such milk results at +0.2 to 0.4 unit higher pH than normal milk. This might be the result of an abundant number and smaller size of the soluble WP/*κ*-Cn complexes that are more likely to condensate on casein micelles, thus accelerating gelation [15, 36, 51]. Another consequence of such complex formation is the reduction of electrostatic repulsion and/or reinforcing hydrophobic attraction among the casein micelles that leads to set gel at relatively higher pH [62].

## 4. Effect of Whey Protein/*κ*-Casein Complexes on Yoghurt Properties

Usually, the micelle-bound denatured whey protein and/or the soluble WP/*κ*-Cn complexes have an effect on the firmness, water holding capacity, whey separation, and rheological and microstructural properties of the yoghurt gels. A comprehensive number of studies have been performed to understand the role of casein micelles associated and WP/*κ*-Cn complexes on the acid gelation and yoghurt texture. Some authors emphasize the role of micelle-bound whey protein complex on the yoghurt texture [10, 60, 63], whereas others highlight the significant role of the soluble WP/*κ*-Cn [64]. A brief overview is presented in Table 1.

### 4.1. Firmness and Water Holding Capacity

Heating milk before fermentation increases the water holding capacity as well as firmness of the yoghurt. These positive effects are correlated with the degree of denaturation of the whey protein and formation of the micelle-bound and/or the soluble WP/*κ*-Cn complexes [14, 57, 62]. Out of these two complexes, there is some evidence that the positive effect of the soluble WP/*κ*-Cn complexes is predominant to raise the water holding capacity and firmness of yoghurt gel than casein micellar complexes on the final firmness and water holding capacity of the acid gel [54–56], whereas others also reported that micelle-bound whey proteins are responsible for increasing the final firmness of acid gels [28, 65]. Regarding the role of micelle-bound whey protein, [65] applied heat treatment on the unheated casein micelles in the presence of soluble denatured WP/*κ*-Cn complexes to increase the micelle-bound complexes and consequently noticed a uniform firmer acid gel. Micellar WP/*κ*-Cn complexes induce the lowering of syneresis or increase water holding capacity due to the high water-binding capacity of the unfolded whey proteins [66]. Other research conversely presented that the above approaches of the micelle-bound and the soluble WP/*κ*-Cn complexes are not the only key factor to consider for changing the gelation properties and/or textural properties of heated skim milk. The total amount of the denatured WP/*κ*-Cn complexes may alter the gelation properties of the heated milk [28, 67, 68]. However, in our recent work, we were able to demonstrate that at normal milk pH, combined effects of micelle-bound and soluble WP/*κ*-Cn complexes increase the firmness and water holding capacity of set type yoghurt [5]. Heat-induced complex formation enhances the formation of covalent disulfide bonds and hydrophobic interaction resulting in an increased connection and compact protein network which ultimately immobilizes more water inside the gel structure [6, 69].

### 4.2. Rheological Properties of Yoghurt

Usually, large or low amplitude rheology has been applied in most studies to understand how storage modulus (*G*′) and reduced tan *δ* of acid milk gels are influenced by the type of WP/*κ*-Cn complexes. The formation of the disulfide bond between denatured whey protein and casein increases the *G*′ and lower tan *δ* [10, 36]. [68] also reported that during fermentation, the formation of the -SH/S-S interchange reaction in the acid gel contributes to a higher *G*′ of acid gel. However, it is difficult to distinguish the specific contribution of the micelle-bound and the soluble WP/*κ*-Cn complexes on the gel strength [6].

Besides, the variations of the mechanical and structural properties of the resulting curd are also studied by altering the heating condition of milk to achieved different proportions of micelle bond and soluble WP/*κ*-Cn complexes [6, 9]. Some studies focused on the effect of micelle-bound complexes by lowering the milk pH < natural milk pH (~6.7) and reported that micelle-bound complexes have a significant role on the final elastic properties of curd [9, 70], whereas other studies demonstrated the important role of the soluble WP/*κ*-Cn complexes on the structural properties of acid gels [69, 71]. Possible reasons for the incompatible results are related to either method of acidifications or the use of different starting materials [5, 72]. However, milk heated at natural pH results in curd with higher *G*′ and lower tan delta due to a balanced contribution of both complexes [6]. Despite the type of protein complexes, the nature of the interactions plays a role to modulate the gel structure. For instance, at high milk pH (>6.7), covalent disulfide bond is dominant and results in higher elastic modulus of the final acid gels [28, 48, 71]. Alternatively blocking the free thiol groups of whey protein by N-ehtyl-maleimide (NEM) results in lower *G*′ due to the absence of disulfide bonds [73].

### 4.3. Yoghurt Microstructure

A uniform microstructure of yoghurt is essential for the texture and the mouth feeling, which are the important parameters for consumer acceptance [74]. Yogurts made from unheated and heated milk shows different microstructural properties. Generally, a more open network with large porous structures is observed in yogurt from unheated milk, whereas a more compact protein network with less porous space is observed in yogurt from heated milk. During heat treatment, milk protein complexes are formed due to covalent bond and hydrophobic interactions, resulting in improve microstructure properties of yogurt [10]. The presence of more aggregating complexes between denatured WP and *κ*-Cn either in the casein micelles or in the serum phase is responsible for increasing the compactness of the network. Some authors also reported that there was no major microstructural difference observed in yogurt gels with various levels of WP/*κ*-Cn complexes [9, 36], while others also reported that a high level of soluble and/or in a combination of micellar WP/*κ*-Cn complexes produce inhomogeneous structures and larger pores in acid gel whereas gel having only micellar WP/*κ*-Cn complexes shows a more homogenous structure with small pores [68, 75].

**Figure 2:**
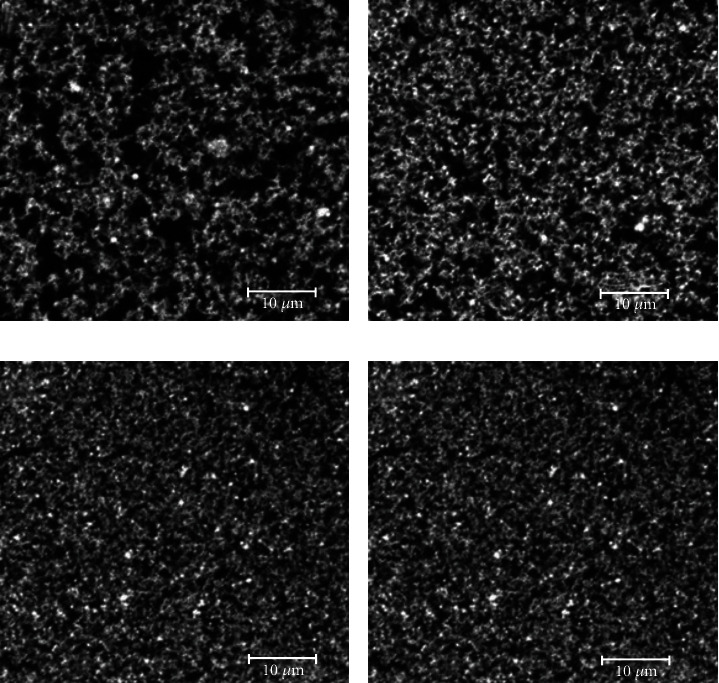
Microscopic images of yoghurt gel made from whey protein enriched skim milk. The protein matrix is grey-white, and the pores are dark. (a) unheated; (b) heated milk at pH 6.3; (c) heated milk at pH 6.7; (d) heated milk at pH 7.1 [6].

In contrast, our result demonstrated that major microstructural differences are observed as a result of the predominant level of micelle-bound or soluble WP/*κ*-Cn complexes present in heat milk (Figure 2) but the combined contribution of both complexes results in a more compact protein network with a less porous microstructure of yogurt. [6]. Possible reasons for the conflicting results are (a) the use of different materials, (b) the application of different methods during acidification, and (c) the fact that most soluble WP/*κ*-Cn should ultimately be combined with micellar caseins at lower pH during acidification. Moreover, the method of acidification (e.g., glucono-*δ*-lactone or lactic acid bacteria) have a significant effect on the microstructural properties of yoghurt gel [4, 14, 63, 71].

## 5. Conclusions

Heat-induced denatured whey protein-casein complexes have an important role in the formation of yoghurt gel structure. A better understanding of the denatured WP/*κ*-Cn complex formation, preferential localization into either micelle bound, or serum phases will facilitate the best utilization of their functionality. A number of studies investigated the formation of these protein complexes in heated milk, and the effects of modulating *κ*-Cn complexes in heated ultimate gel properties are well reported. Based on the existing knowledge of casein micelle complex formation, future trend of casein research might lead us to focus on constructing artificial casein (e.g., microbial origin) or casein complex formation with nondairy proteins (e.g., plant protein) as alternative ingredients for yoghurt.

## Figures and Tables

**Table 1:** Possible complex formation in heated milk and their effect on gel properties.

System condition	Factors	Protein complex	Influence on get properties	Reference
Reconstituted skim (10.7% TS) and added WP heated at 80°C for 30 min	pH at heating (6.2-7.2)	MBC and SPSPC	pH > natural milk pH, mostly soluble protein pH < natural milk pH mostly micelles bound complexes At natural pH both complexes were contributed Gel firmness, *G*′ and WHC were higher at natural pH than other pH at heating of milk Interconnected dense protein network was observed at natural pH at heating	[5] [6] [9] [71]
Reconstituted milk (4% protein) heated at 95°C for 5 min	CN : WP ratio (4 : 1 to 1 : 1)	S-S bond and hydrophobic interactions	*G*′ yields stress and strain enhanced with decreasing CN : WP	[10]
Yak milk, heated at 85°C for 10 min	pH at heating (6.6-7.4)	MBC and SPSPC	Soluble complex is predominant for increasing *G*′, WHC and firmness	[56]
Standardized milk heated at 95°C for 4 min	CN : WP ratio (4 : 1 to 0.6 : 1)	Self-aggregation of WP and aggregated WP-coated fat globules	Particle size, firmness, and apparent viscosity increased as a function of WP content Interrupted and coarse gel observed in presence of large WP aggregates and lower number of fat globules	[75]
Reconstituted milk (3.5% protein) heated at 90°C for 13 min	Heating condition of milk	MBC and SPSPC	Firm gel only in presence of heat induced complexes (casein micelles or serum or both) Rheological and microstructural properties were similar for acid gel containing MBC and SPSPC	[57]
Reconstituted milk (10.45% TS) heated at 80°C for 10 min	pH at heating (6.2-6.9)	S-S bond and soluble WP aggregates	The *G*′ and *G*^″^ value increase as function of pH of heating milk	[69]
Fresh milk heated at 85°C for 3 min	Heating condition and pH during gelation	MBC and SPSPC	WP/*κ*-casein complexes cause casein micelles (heated/unheated) to aggregates at higher pH Denatured serum protein and native casein micelles interacts directly at pH > 5.2	[28]
Reconstituted milk (10% TS) heated at 90°C for 10 min	Milk pH at heating (6.5-7.2)	Soluble protein complex formation in serum phase	Stronger gel with increased amount of soluble protein complex More soluble protein complex at higher milk pH at heating	[64]
Reconstituted milk (11% TS) sodium caseinate 0.5-1%	Heating condition and sodium caseinate	MBC and SPSPC	Without sodium caseinate mostly soluble WP/*κ*-casein complexes sodium caseinate mostly bond with the surface layer of *κ*-casein	[48]
Reconstituted milk (12% TS) heated at 82.5°C for 10 min	Inoculation rate (0.1-4%)	Hydrophobic interaction, interconnected or branched protein networks	Higher *G*′, yield stress and WHC as function of inoculation rate Smaller pore size and more interconnected gel network	[74]
Reconstituted milk (4.7% protein) heated at 95°C for 10 min	CN : WP ratio (4 : 1 to 1 : 1)	MBC and SPSPC	*β*-Lac and *α*-lac interacted with *κ*-casein and *α*_s2_-casein Some portion of *κ*-casein still remained unreacted after heating	[55]
Casein/whey protein dispersions heated at 90°C for 20 min	Type of whey protein in the mixture (heated or unheated)	WP aggregates and WP-coated casein micelles	Higher gel hardness due to denatured whey proteins aggregates Gel hardness and *G*′ increased due disulfide bond among protein complexes	[67, 68]
Reconstituted milk (12% TS) heated at 85°C for 10 min	CN : WP ratio (4.7 : 1 to 0.5 : 1)	MBC and SPSPC	Gel strength, WHC and finer crosslinked structure obtained with decreasing CN : WP ratio	[66]
Casein/WP dispersions heated for 20 min	Heating condition of milk	Casein/WP complex	Unheated milk, gel formation was due to casein micelle aggregation Above 60°C firmer gel was due to whey protein/*κ*-casein complex	[65]
Reconstituted milk (12% TS) heated at 85°C for 5 min	Type of acidification (GDL or bacterial)	MBC	Acidification with GDL resulted in lower *G*′ higher *δ*, higher permeability Acidification with bacteria resulted in higher *G*′, low *δ* and lower permeability	[63]

*G*′: storage modulus; *G*^″^: loss modulus; MBC: micelle-bound complexes (WP/*κ*-casein complexes); SPSPC: serum phase soluble protein complexes (WP/*κ*-casein complexes); S-S: disulfide; GDL: glucono-*δ*-lactone; CN: casein; WP: whey protein; WHC: water holding capacity.

## Data Availability

Data used in this manuscript are available upon request to corresponding author.
